# Plasma Lipidomics Profiling of Developmental Dysplasia of the Hip in Tibet Plateau

**DOI:** 10.1002/hcs2.70012

**Published:** 2025-04-06

**Authors:** Xiaogang Li, Jiamei Ji, Ping Li, De Yang, Nyima Yedron, Yanming Lei, Tao Chen, Jianchu Li, Ye Guo, Xiao Yang, Li Shi, Dan Qu

**Affiliations:** ^1^ Biobank Facility, National Infrastructures for Translational Medicine, State Key Laboratory of Complex Severe and Rare Diseases Peking Union Medical College Hospital, Chinese Academy of Medical Science and Peking Union Medical College Beijing China; ^2^ Department of Ultrasound, State Key Laboratory of Complex Severe and Rare Diseases, Peking Union Medical College Hospital Chinese Academy of Medical Science and Peking Union Medical College Beijing China; ^3^ Department of Orthopedics People's Hospital of Tibet Autonomous Region Lhasa Tibet China; ^4^ Department of Ultrasound People's Hospital of Tibet Autonomous Region Lhasa Tibet China; ^5^ Department of Radiology People's Hospital of Tibet Autonomous Region Lhasa Tibet China; ^6^ Department of Ultrasound, Beijing Jishuitan Hospital, The 4th Clinical College Peking University Beijing China; ^7^ State Key Laboratory of Complex Severe and Rare Diseases, Department of Clinical Laboratory, Peking Union Medical College Hospital Chinese Academy of Medical Science and Peking Union Medical College Beijing China; ^8^ Department of Infectious Diseases People's Hospital of Tibet Autonomous Region Lhasa Tibet China; ^9^ Department of Laboratory Medicine People's Hospital of Tibet Autonomous Region Lhasa Tibet China

**Keywords:** developmental dysplasia of the hip, diagnosis, lipidomics, pediatric orthopedic, phosphatidylethanolamine, triacylglycerols

## Abstract

**Background:**

Developmental dysplasia of the hip (DDH) is a prevalent pediatric condition with a multifactorial etiology. Its incidence varies geographically, with notably higher rates observed on the Tibet plateau. This study was performed to evaluate the lipidomics signatures associated with DDH by analyzing plasma samples.

**Methods:**

Fifty infants were recruited, including 25 diagnosed with DDH and 25 age‐matched healthy controls. In addition to plasma samples, comprehensive laboratory test results and medical records were collected for each participant. An untargeted lipidomics profiling approach was employed to identify distinguishing metabolic signatures.

**Results:**

Lipidomics profiles differed significantly between patients with DDH and healthy controls. Several differential metabolites were identified, including triacylglycerol (TAG)(17:0/18:1/20:1), TAG(17:0/17:0/17:0), phosphatidylethanolamine (PE)(10:0/26:4), TAG(17:0/18:0/18:0), TAG(16:0/17:0/22:1), TAG(16:0/18:0/22:0), TAG(17:0/19:0/19:0), TAG(13:0/20:0/20:0), TAG(18:0/18:0/22:0), and TAG(16:0/20:0/20:0). The primary lipid species showing differences were TAGs and PE.

**Conclusions:**

Distinct shifts in lipidomics profiles were observed in infants with DDH. To the best of our knowledge, this study is the first to explore lipidomics signatures in patients with DDH. The combined assessment of TAG(17:0/18:1/20:1) and TAG(17:0/17:0/17:0) may serve as a potential diagnostic tool for DDH.

## Graphical abstract

Overall workflow of this study.

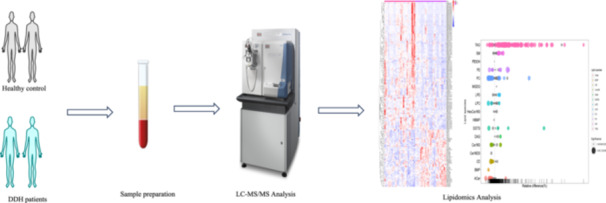

**Abbreviations**
DDH: developmental dysplasia of the hip
MS: mass spectrometry
OPLS‐DA: orthogonal partial least‐squares discriminant analysis
PE: phosphatidylethanolamine
TAG: triacylglycerol
UPLC: ultra‐performance liquid chromatography
VIP: variable importance in projection

## 1 Introduction

Developmental dysplasia of the hip (DDH) is the most common hip joint disorder in pediatric orthopedics, encompassing conditions such as hip dislocation, subluxation, and acetabular dysplasia [1, 2]. A previous study assessed the hip joints of 606 infants in Tibet using ultrasound and other examination methods. The findings revealed a DDH prevalence of 1.81% among infants in Tibet, with a significant positive correlation between prevalence and altitude. Specifically, for every 1000‐m increase in altitude, the prevalence of DDH increased by 0.56% (*p* = 0.004). These results indicate that the prevalence of DDH in infants in Tibet is relatively high and is positively associated with altitude [2].

DDH is a complex disorder encompassing a spectrum of hip abnormalities, ranging from neonatal instability to acetabular or femoral dysplasia, hip subluxation, and complete dislocation. It can present during the neonatal period, with most cases of hip instability resolving spontaneously within the first few weeks of life. While the exact etiology remains unclear, several risk factors have been identified, including breech presentation (particularly in female infants), the presence of other deformities such as torticollis or congenital foot abnormalities, and a family history of DDH.

The management of DDH requires a multidisciplinary approach involving pediatricians, orthopedic surgeons, and physiotherapists. Regular follow‐up is crucial to monitoring hip development and adjusting treatment as needed. If left untreated, DDH can lead to severe complications, including chronic pain, deformity, and early‐onset degenerative joint disease, potentially resulting in long‐term disability.

Early detection and treatment are essential for improving the quality of life in children with DDH. The primary goal of treatment is to achieve concentric reduction of the femoral head and facilitate normal development of the acetabulum and proximal femur. Overall, DDH is a complex condition that requires prompt detection and appropriate management to prevent long‐term complications. Understanding its etiology, recognizing clinical symptoms, and implementing timely therapeutic interventions are crucial for improving patient outcomes.

Prompt detection of anomalies through early screening, followed by immediate noninvasive corrective measures, can significantly reduce the likelihood of prolonged hip replacement procedures and lower long‐term disability rates [3, 4]. The Standing Medical Advisory Committee recommends clinical screening for DDH in all neonates. This comprehensive screening regimen includes evaluations at birth, upon discharge, at 6 weeks, between 6 and 9 months, and after the onset of ambulation. Ultrasound screening has become an invaluable tool for early diagnosis, providing a detailed assessment of hip joint morphology, femoral head positioning, and overall hip stability. Among various techniques, the Graf method—which evaluates the hip joint's coronal section via ultrasound—remains both pioneering and the most effective diagnostic tool [5, 6].

Nevertheless, the complexities of ultrasound screening require a high level of professional expertise, contributing to the risk of both misdiagnoses and missed diagnoses. The challenge of early detection is further exacerbated by the subtle clinical manifestations and often indistinct initial symptoms of DDH, highlighting the urgent need for biomarkers to enhance diagnostic accuracy. Metabolomics, which enables the comprehensive analysis of metabolites with molecular masses of < 1000 in clinical specimens, holds promise for identifying molecular patterns associated with pathological changes [7, 8, 9, 10]. This approach has been instrumental in developing biomarkers for early disease detection and therapeutic monitoring. A novel study by Rhodes et al. investigated biochemical pathways involved in skeletal development to explore potential links to DDH etiology. The study analyzed spot urine samples from 99 infants—30 with DDH and 69 age‐matched controls—using ion chromatography‐mass spectrometry to quantify thiosulphate, sulfate, nitrate, and phosphate, while nitrite was measured via high‐performance liquid chromatography. Significant differences were observed in thiosulphate, TBARS, and creatinine concentrations between the DDH group and controls (*p* = 0.025, 0.015, and 0.004, respectively), and urine osmolality was significantly lower in DDH infants (*p* = 0.036), indicating the production of more diluted urine. In this context, we conducted comprehensive lipidomics profiling to identify specific metabolites closely associated with DDH pathogenesis.

## 2 Materials and Methods

### 2.1 Patients

A total of 25 patients diagnosed with DDH (hereafter referred to as the DDH group) and 25 healthy controls (HC group) were enlisted from the People's Hospital of Tibet Autonomous Region between January 2021 and March 2022. The diagnosis was established by a seasoned clinician based on comprehensive examination findings. To be eligible for the study, participants had to have a verified diagnosis of one of these conditions: (1) a total hip joint dislocation, where there was no contact between the original joint surfaces; (2) a partial hip joint dislocation, with some contact remaining between the joint surfaces; or (3) acetabular dysplasia, marked by underdevelopment of the acetabulum. Experienced pediatric orthopedic experts made the diagnoses after conducting thorough medical assessments. Other clinical signs taken into account included the noticeable upward and outward movement of the greater trochanters, limited hip joint abduction, and positive Ortolani, Barlow, Allis, or Galeazzi signs. X‐ray imaging was used to confirm the diagnoses. The control group was made up of healthy children in Tibet who had regular health check‐ups at the People's Hospital of Tibet and showed no abnormalities.

Fasting blood samples were drawn using venipuncture into Vacuette tubes prefilled with a procoagulant. Within a frame of 15–30 min post‐collection, the samples underwent centrifugation at 1200×g for a duration of 10 min. The clinical data and hospital records pertaining to all participants are delineated in Table 1. Biospecimens from patients were collected following approval from the People's Hospital of Tibet Autonomous Region institutional committee tasked with protecting human subjects (ME‐TBHP‐21‐023). Informed consent was procured from each participant subsequent to thorough clarification regarding the procedures and intent of the study.

**Table 1:** Clinical characteristics of patients with developmental dysplasia of the hip (DDH).

	*N* = 25
Age (months)	19.9 ± 9.1
Mean altitude of residence (m, mean ± SD)	4101.9 ± 344.7
Gender (female/male)	(18/7)
Oligohydramnios, *n* (%)	2 (8)
Post‐term birth, *n* (%)	1 (4)
Vaginal delivery, *n* (%)	23 (92)
First delivery, *n* (%)	4 (16)
Birth weight (kg, mean ± SD)	3.5 ± 0.5
Neonatal macrosomia, *n* (%)	9 (36)
Tight swaddled or twined baby wraps, *n* (%)	11 (44)
Affected hip joint(s) (left/right/bilateral)	6/6/13
Underwent open surgical treatment, *n* (%)	22 (88)

### 2.2 Materials and Equipment

Reagents such as mass spectrometry grade ammonium formate and dichloromethane were procured from Fisher Chemical, while mass spectrometry grade methanol and acetonitrile were sourced from CNW Technologies. For lipidomics profiling, we employed a liquid chromatography system (1290, Agilent Technologies). interfaced with the Q Exactive Focus (Thermo Fisher Scientific). Separation was achieved using the ACQUITY UPLC BEH Amide column (1.7 μm, 2.1 × 100 mm, Phenomen).

### 2.3 Sample Preparation

For the lipidomics assessment, 100 μL of each specimen was amalgamated with 480 μL of a methanol/MTBE solution (mixed in a 1:5 volume ratio). This amalgamation took place in a sterile EP tube. Subsequent to vigorous vortexing, the samples were sonicated for 10 min in an ice‐water bath. Post incubation of 1 h at −40°C, the samples were subjected to centrifugation at 3000 rpm for 15 min at 4°C. The supernatant was then earmarked for mass spectrometry analysis. It should be noted that the quality control (QC) sample was constituted by pooling an equal volume 20 μL from all the individual samples.

### 2.4 Lipidomics Analysis

MS analyses were carried out using an UPLC system (1290, Agilent Technologies) coupled to an Q Exactive Focus (Thermo Fisher Scientific) operating in data‐dependent acquisition (DDA) mode. The separation was achieved using the ACQUITY UPLC BEH Amide column (1.7 μm, 2.1 × 100 mm, Phenomen). The mobile phase comprised 10 mmol/L ammonium formate and 60% acetonitrile in water (pH = 9.75) (A), and 10% acetonitrile in isopropanol (B). The autosampler was maintained at 4°C with an injection volume set at 2 μL. ESI source conditions were as follows: capillary temperature at 320°C, spray voltage at 5 kV (positive) or −4.5 kV (negative), sheath gas flow rate at 30 Arb, auxiliary gas flow rate at 10 Arb, full MS resolution at 70,000, MS/MS resolution at 17,500, collision energy at 15/30/45 in NCE mode.

### 2.5 Data Processing

For lipidomics analysis, raw MS data were transformed with ProteoWizard software to mzXML. Initially, we conducted a range of data management tasks, which comprised the following steps: (1) filtering out off‐values, (2) eliminating missing values, (3) imputing missing values, and (4) normalizing the data. In this process, the internal standard (IS) was employed for the normalization step. Selecting the ion mode is an important step in metabolomics, mainly based on the properties of compounds and the mobile phase environment. In our project, we used both modes. Tasks such as peak picking, peak extraction, alignment, and integration were accomplished using the CentWave algorithm in XCMS software, identification was accomplished using LipidBlast library.

### 2.6 Statistical Analysis

Data are presented as mean ± SD. Statistical evaluations were conducted using the R package (ropls). Within the orthogonal partial least‐squares discriminant analysis (OPLS‐DA) model, the variable importance in the projection (VIP) value for each parameter was computed. Metabolites with a VIP value exceeding 1 were further assessed for significance using the Student's *t*‐test at the univariate level. A *p*‐value of less than 0.05 was deemed statistically significant. The heatmap and receiver operating characteristic (ROC) analyses were generated utilizing MetaboAnalyst 5.0 (Xia Lab @ McGill, Sweden).

## 3 Results

### 3.1 Clinical Data of DDH Patients

All participants in the study were of Tibetan ethnicity (100%). The average age of the children enrolled was 19.9 ± 9.1 months. The residence altitude was recorded as 4101.9 ± 344.7 meters. None of the participants had a family history of developmental dysplasia of the hip (DDH). The gender ratio was 18 girls to 7 boys. During the fetal period, there were two cases (8%) of oligohydramnios, one case (4%) of post‐term birth, and all deliveries (92%) were vaginal with cephalic presentations. Four cases (16%) represented primiparous mothers. The average birth weight was 3.5 ± 0.5 kg, with nine infants (36%) weighing more than 4 kg at birth. Swaddling with tight wrappings, such as candle wraps, was practiced in eleven cases during infancy (44%). Among the total number of children evaluated, seven had left hip involvement, eight had right hip involvement, and eleven had bilateral hip involvement respectively. Open surgical treatment was performed in twenty‐two cases (88%), following diagnosis (Table 1).

### 3.2 Raw Data Preprocessing

To mitigate the influence of detection system errors and enhance the biological relevance of our findings, we performed rigorous data management on the primary data set. This process encompassed several steps:

1. Off‐value Filtering: We filtered individual deviation peaks to eliminate noise, using the relative standard deviation (RSD) as a criteria.
2. Missing Value Filtering: We retained only the peak area data if they had no more than 50% null value in a single group or no more than 50% hollow value across all groups.
3. Imputation of Missing Values: Missing data points in the original data set were rectified. We employed a numerical simulation method, imputing with half of the observed minimum value.
4. Data Normalization: Data normalization was achieved using an internal standard (IS).

### 3.3 Cluster Analysis (OPLS‐DA)

Given the high‐dimensional nature of the metabolomic data (with numerous detected metabolites) and the limited sample size, we utilized OPLS‐DA. This analytical method allowed us to exclude orthogonal variables in metabolites, which are not related to categorical distinctions, providing a more accurate depiction of variations among metabolite groups and the correlation magnitude of the test group.

**Figure 1:**
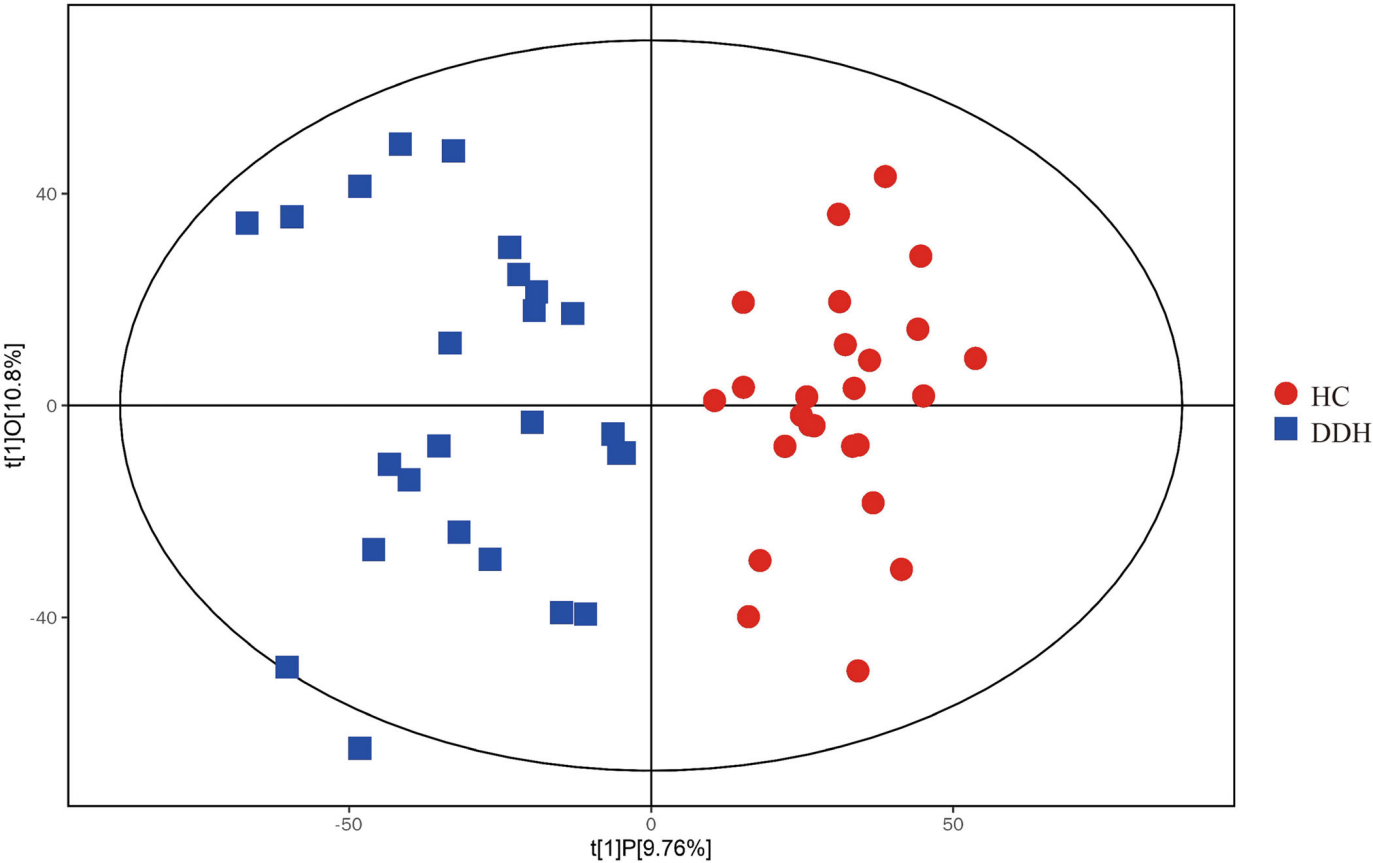
OPLS‐DA model of lipidomics in ESI+/ESI− ionization mode. ESI+, electrospray ionization in positive mode; ESI−, electrospray ionization in negative mode.

In the provided figure, the horizontal axis t[1]P signifies the predicted primary component score of the first principal component, highlighting intergroup sample differences. The vertical axis t[1]O denotes the orthogonal primary component score, underscoring intra‐group sample variations. The results from the OPLS‐DA score plot indicate a significant distinction between the two sample groups, with most samples falling within a 95% confidence interval (Figure 1).

### 3.4 Volcano Map

**Figure 2:**
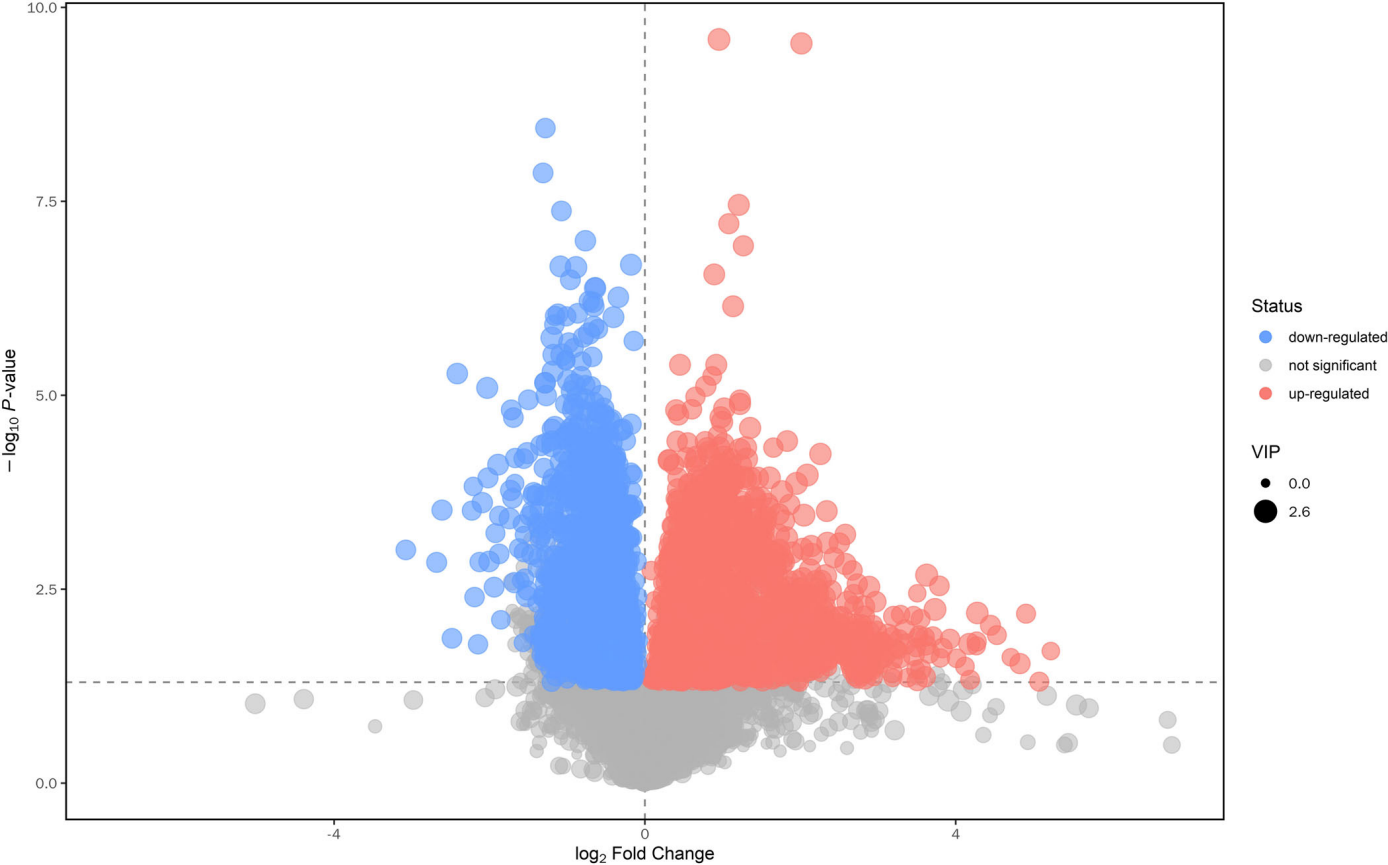
Volcano plot of lipidomics in ESI+ ionization mode.

The volcano map provides a visual representation of the overall distribution of differential metabolite levels between the groups. The two primary indicators: a *p*‐value from the student's *t*‐test less than 0.05 and a Variable Importance in the OPLS‐DA Model greater than 1. In this map, each point symbolizes a peak. Metabolites that are significantly upregulated are depicted in red, downregulated metabolites are in blue, and those without significant differentiation are in gray (as shown in Figure 2).

### 3.5 Heatmap

**Figure 3:**
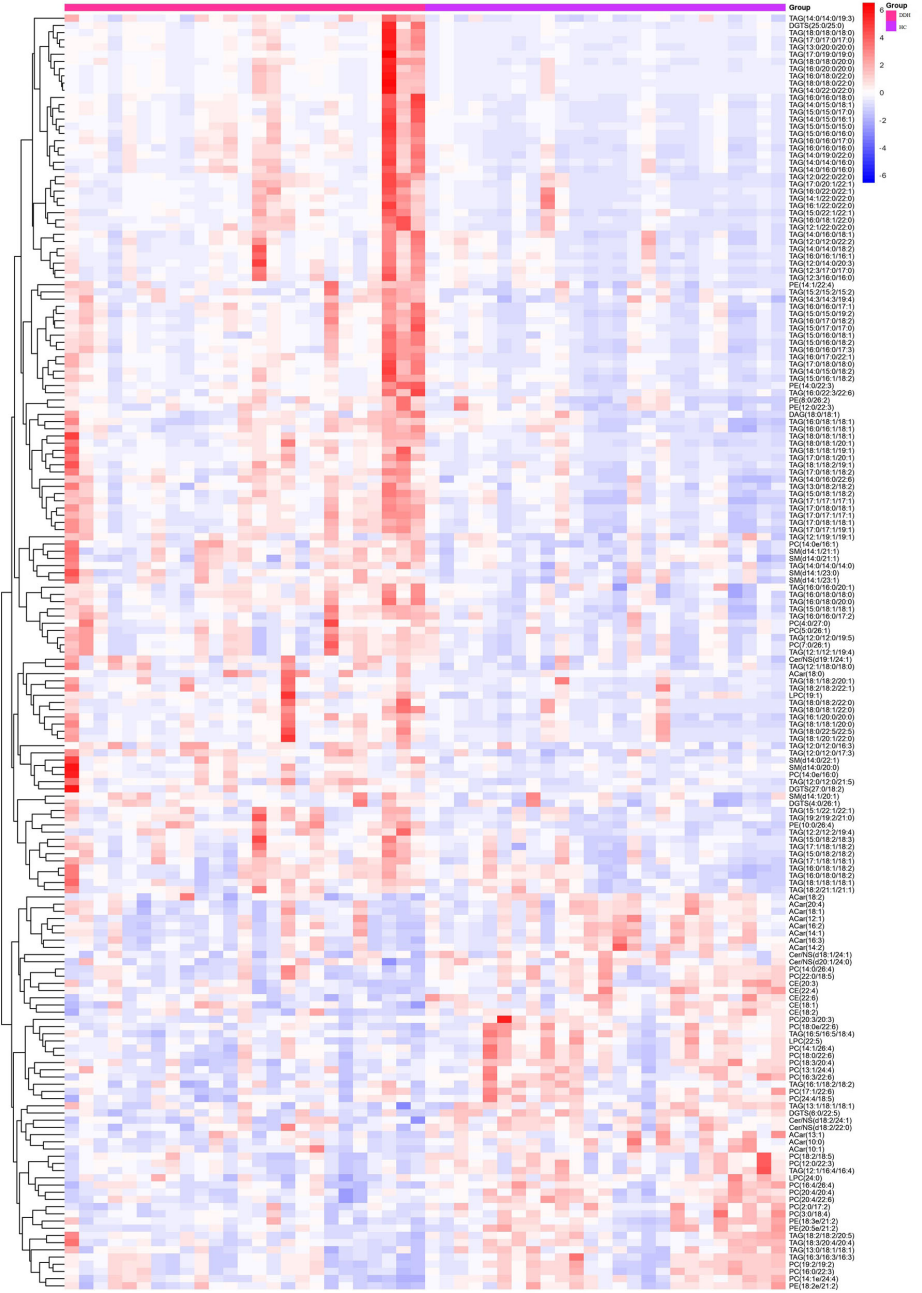
Heatmap of the top differentially expressed features in lipidomics.

The heatmap was constructed through the quantitative values of differential metabolites by calculating the Euclidean distance matrix. A complete linkage clustering method was employed to cluster these metabolites. In the heatmap, the *x*‐axis represents the different experimental groups while the *y*‐axis denotes the varied metabolites being compared within the groups. The color coding within the heatmap (as shown in Figure 3) corresponds to the relative expression levels of the metabolites, with red indicating higher expression and blue suggesting lower expression.

### 3.6 Matchstick Diagram and Radar Chart Analysis

**Figure 4:**
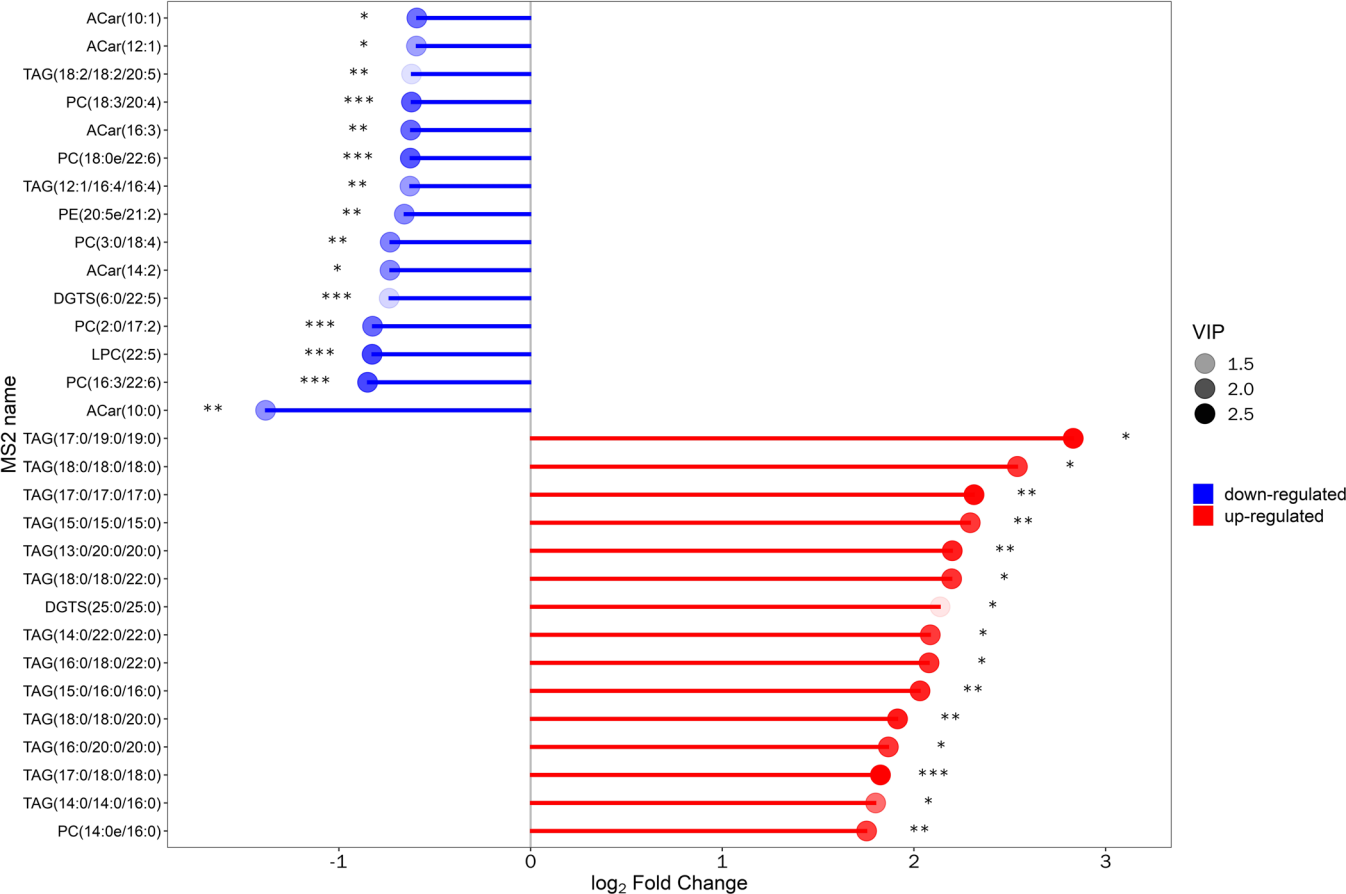
Matchstick diagram comparing DDH versus control groups.

We determined the corresponding ratio for the differential metabolites’ quantitative values, selecting the top 15 for both upregulation and downregulation to present the results. In the diagram, changes in multiple are illustrated on the *x*‐axis, while the intensity of the dot color indicates the magnitude of the VIP value. This analysis highlights metabolites exhibiting substantial alterations, suggesting that the expression level of their associated enzymes could be either significantly inhibited or activated (*0.01 < *p* < 0.05, **0.001 < *p* < 0.01, ***p < 0.001). Further validation can be conducted to examine the regulatory impact on enzyme gene expression by certain metabolites pertinent to the study (as depicted in Figure 4).

**Figure 5:**
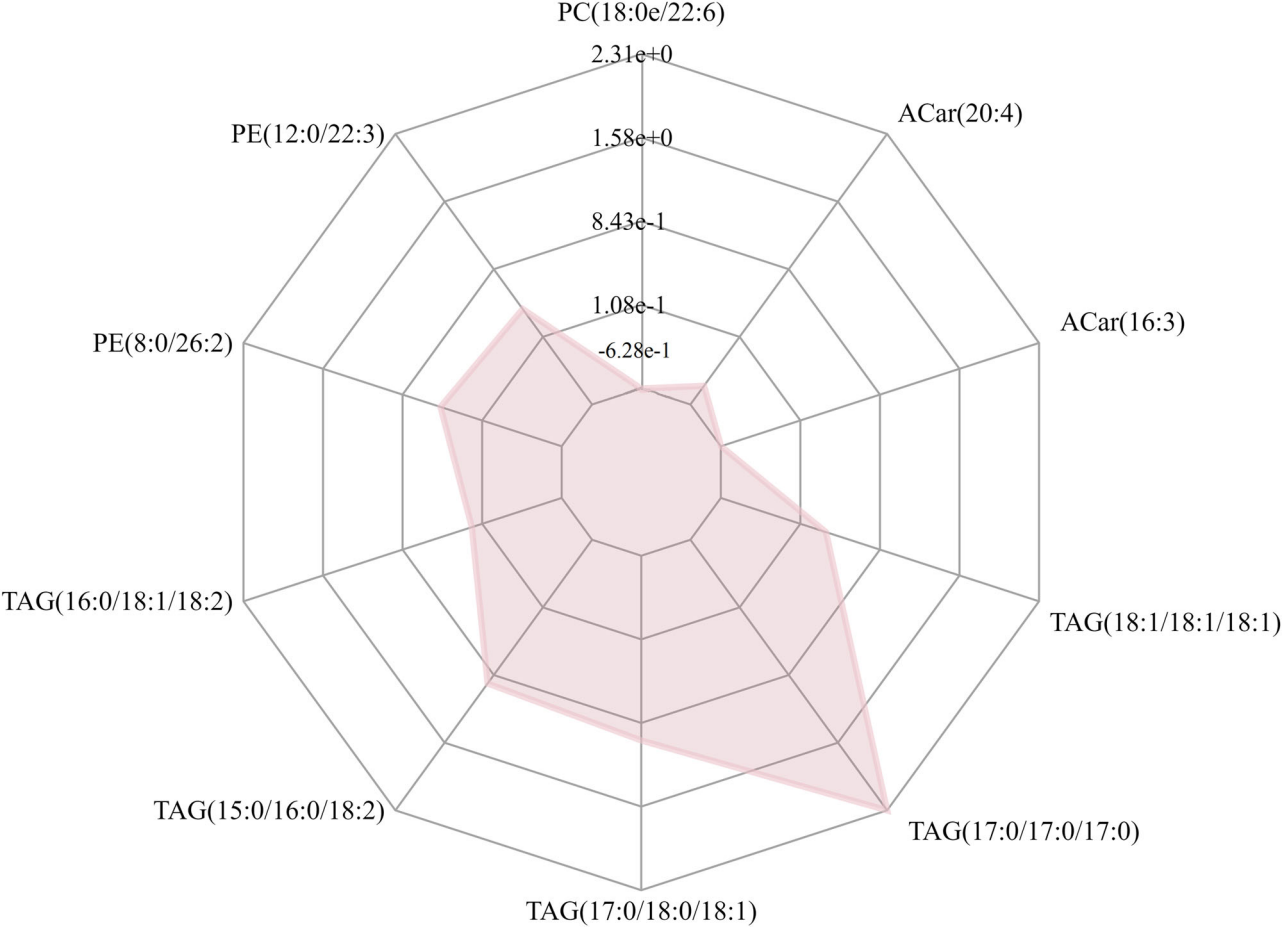
Radar chart analysis for the DDH versus control groups.

For the radar chart, we again computed the corresponding ratio for the differential metabolite's quantitative values and applied a logarithmic transformation with a base of 2. In the chart, purple shadows are formed by the lines indicating the difference multiples for each metabolite, showcasing the evolving trend of content in the radar map (refer to Figure 5).

### 3.7 Bubble Chart

**Figure 6:**
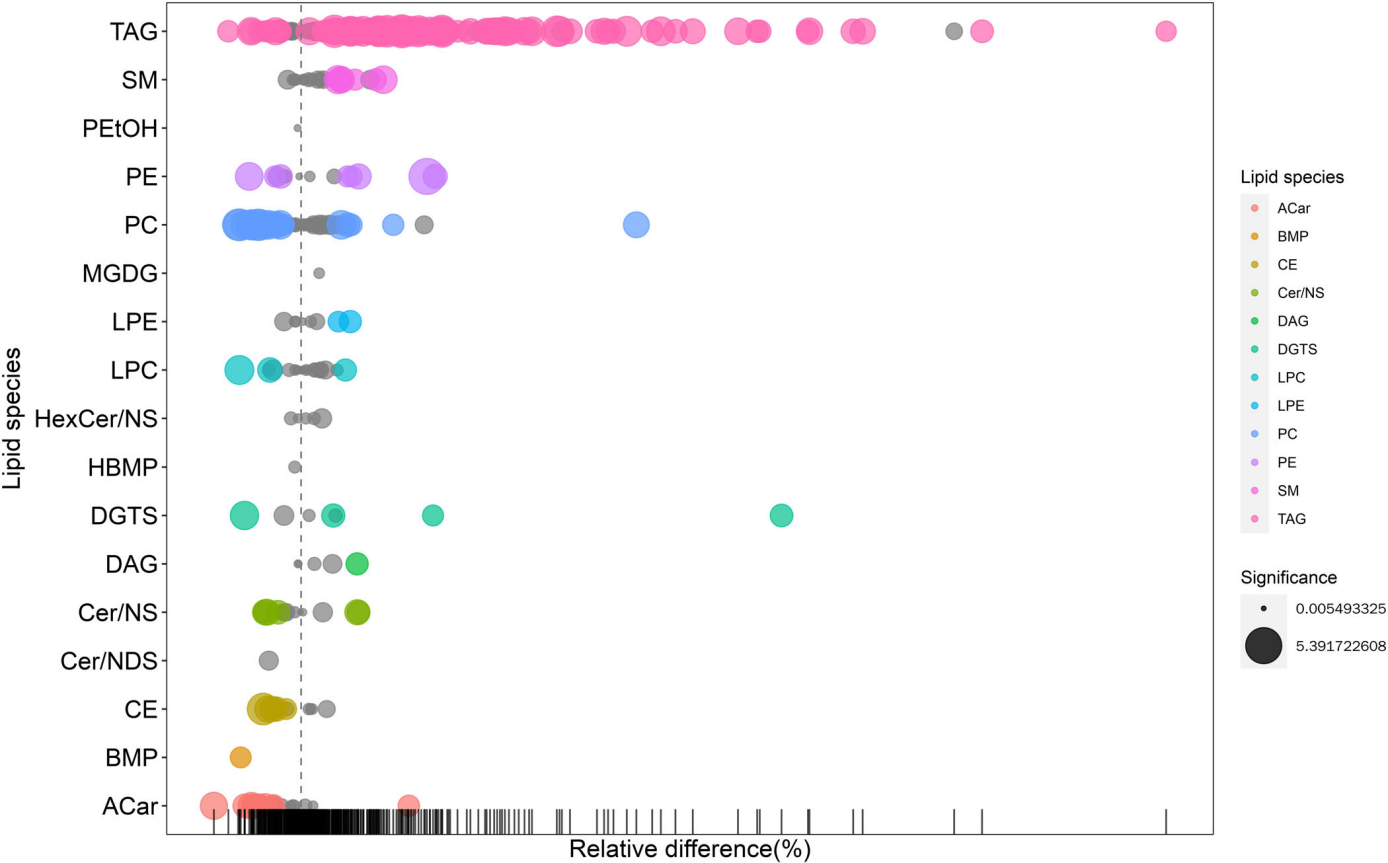
Bubble plot comparing DDH versus control groups.

Our metabolic pathway analysis results are visually represented in a bubble chart. The bubble's horizontal size and position demonstrate the pathway's influence factor based on topological analysis. A darker bubble shade indicates a smaller *p*‐value, denoting a higher enrichment significance. The main lipid species that showed differences include primary triacylglycerols (TAGs) and phosphatidylethanolamine (PE). In the field of bioinformatics, the area under the curve (AUC) is frequently utilized to assess the effectiveness of classification models. Ranging from 0 to 1, this metric gauges the model's capacity to categorize samples. Typically, the closer the AUC is to 1, the greater the reliability of the detection method. Conversely, an AUC of 0.5 indicates the lowest reliability and lacks practical value. In this study, we examined the AUC of various metabolites (see Figure 6, Table 2). TAG (17:0/18:1/20:1) and TAG (17:0/17:0/17:0) have the potential to become valuable diagnostic tools in the future.

**Table 2:** AUC of different metabolites.

MS name	AUC	CI (95%)
TAG (17:0/18:1/20:1)	0.917	0.836–0.998
TAG (17:0/17:0/17:0)	0.912	0.826–0.998
PE (10:0/26:4)	0.901	0.819–0.983
TAG (17:0/18:0/18:0)	0.896	0.803–0.989
TAG (16:0/17:0/22:1)	0.894	0.808–0.981
TAG (16:0/18:0/22:0)	0.891	0.799–0.984
TAG (17:0/19:0/19:0)	0.891	0.802–0.981
TAG (13:0/20:0/20:0)	0.886	0.792–0.981
TAG (18:0/18:0/22:0)	0.886	0.787–0.986
TAG (16:0/20:0/20:0)	0.880	0.784–0.976

Abbreviations: AUC, area under the curve; CI, confidence interval; MS, mass spectrometry; PE, phosphatidylethanolamine; TAG, triacylglycerol.

## 4 Discussion

DDH stands out as the predominant hip joint disorder in pediatric orthopedics, encompassing hip dislocation, subluxation, and acetabular dysplasia [8, 11, 12]. It is imperative to initiate early treatment for DDH to achieve a hip morphology that is normal or closely approximates normality. The Graf method of ultrasound examination offers the earliest technique to assess the coronal section of the hip joint. Presently, it is lauded as the most efficacious method for both screening and diagnosis. However, the efficacy of ultrasound screening is contingent on high technical expertise, and there remains a measurable rate of both misdiagnosis and under‐diagnosis [7, 13]. Given the subtle clinical presentations and mild preliminary symptoms of DDH, early diagnosis poses challenges. To address this, our research turned to untargeted lipidomics to pinpoint potential biomarkers for DDH [14].

In our endeavor, the untargeted lipidomics approach identified potential plasma biomarkers for DDH, among which TAG(17:0/18:1/20:1), TAG(17:0/17:0/17:0), PE(10:0/26:4), TAG(17:0/18:0/18:0), TAG(16:0/17:0/22:1), TAG(16:0/18:0/22:0), TAG(17:0/19:0/19:0), TAG(13:0/20:0/20:0), TAG(18:0/18:0/22:0) and TAG(16:0/20:0/20:0) were the principal differential metabolites. Abnormalities in lipid metabolism, such as the increase of oxidized lipids and elevated plasma cholesterol levels, can affect the homeostasis of the bone microenvironment through interorgan communication. This influence includes promoting the differentiation of mesenchymal stem cells into adipocytes while inhibiting their differentiation into osteoblasts, as well as promoting the secretion of certain cytokines by osteoblasts to stimulate the differentiation of osteoclasts, ultimately affecting the balance of bone metabolism [15].

The main lipid species that showed differences include primary triacylglycerols (TAGs) and phosphatidylethanolamine (PE). The triglyceride glucose (TyG) index is associated with bone mineral density (BMD), and an increase in the TyG index may correlate with lower lumbar spine BMD, indicating that disorders of lipid and glucose metabolism may be related to an increased risk of osteoporosis. Lipid metabolism disorders may promote the secretion of certain cytokines by osteoblasts, such as the receptor activator of nuclear factor‐κB ligand (RANKL), which can stimulate the differentiation of osteoclasts and further affect the balance between bone absorption and formation [16, 17, 18].

DDH is characterized by pathological changes in both the skeletal and soft tissues. These changes include alterations in the osseous structures, such as the acetabulum, femoral head, femoral neck, and femoral shaft, as well as modifications in the soft tissues like the labrum, ligamentum teres, and joint capsule. Additionally, there may be associated dysfunctions in the muscles surrounding the hip joint. Thus, lipid metabolism not only plays an important role in bone development but is also likely to be associated with the occurrence and development of various bone metabolic diseases such as DDH [19]. DDH is a prevalent pediatric hip condition that currently lacks specific biomarkers. In our study, we carried out metabolomic analysis and identified metabolic pathways that are differentially associated with DDH. TAG (17:0/18:1/20:1) and TAG (17:0/17:0/17:0) have the potential to become valuable diagnostic tools in the future.

## 5 Conclusions

In this study, we elucidated the untargeted lipidomics profiles of plasma in DDH patients. The main lipid species that showed differences include primary triacylglycerols (TAGs) and phosphatidylethanolamine (PE). Although there is no direct evidence indicating a direct relationship between the lipidome and DDH, considering the ubiquitous role of lipids in cellular structure and function, we can speculate that abnormalities in lipid metabolism may indirectly affect joint health and development. TAG (17:0/18:1/20:1) and TAG (17:0/17:0/17:0) have the potential to become valuable diagnostic tools in the future. However, the precise underlying mechanisms warrant further exploration. There are some limitations to this study: (1) Given the rarity of DDH, our sample size was limited, reflecting the challenge of assembling a large cohort from a single center. (2) The robustness and reliability of the identified potential biomarkers require further validation.

There is a paucity of research and references in this specific area, highlighting the need for extended investigations to clarify these findings and to identify the exact mechanism behind the observed lipid changes. Further scientific research and literature exploration may be required for a deeper understanding of the connection between the lipidome and joint development.

## Author Contributions

Xiaogang Li, Jiamei Ji, Ping Li, and Xiao Yang were pivotal in the conception and design of the study. Li Shi, Dan Qu, De Yang, Nyima Yedron, and Yanming Lei were instrumental in patient sample and data collection. Xiaogang Li, Jiamei Ji, and Ping Li played a significant role in data acquisition, data analysis, and drafting of the manuscript. Tao Chen, Jianchu Li, Ye Guo, Xiao Yang, Li Shi, and Dan Qu were responsible for revising the manuscript. All authors have reviewed and approved the final version of the manuscript.

## Ethics Statement

Biospecimens from participants were collected following approval from the People's Hospital of Tibet Autonomous Region institutional committee tasked with protecting human subjects (ME‐TBHP‐21‐023).

## Consent

Informed consent was procured from each participant subsequent to thorough clarification regarding the procedures and intent of the study.

## Conflicts of Interest

The authors declare no conflicts of interest.

## Data Availability

The data set supporting the conclusions of this study is encompassed within the manuscript.
